# Importance of sampling frequency when collecting diatoms

**DOI:** 10.1038/srep36950

**Published:** 2016-11-14

**Authors:** Naicheng Wu, Claas Faber, Xiuming Sun, Yueming Qu, Chao Wang, Snjezana Ivetic, Tenna Riis, Uta Ulrich, Nicola Fohrer

**Affiliations:** 1 Department of Hydrology and Water Resources Management, Institute for Natural Resource Conservation, Kiel University, 24118 Kiel, Germany; 2 Aarhus Institute of Advanced Studies, Aarhus University, Høegh-Guldbergs Gade 6B, 8000 Aarhus C, Denmark; 3 Department of Bioscience, Aarhus University, Ole Worms Allé 1, 8000 Aarhus C, Denmark; 4 Pearl River Fisheries Research Institute, Chinese Academy of Fishery Science, 510380 Guangzhou, China; 5 Marine Science and Engineering, Red Sea Research Centre, King Abdullah University of Science and Technology, Thuwal 23955-6900, Saudi Arabia

## Abstract

There has been increasing interest in diatom-based bio-assessment but we still lack a comprehensive understanding of how to capture diatoms’ temporal dynamics with an appropriate sampling frequency (ASF). To cover this research gap, we collected and analyzed daily riverine diatom samples over a 1-year period (25 April 2013–30 April 2014) at the outlet of a German lowland river. The samples were classified into five clusters (1–5) by a Kohonen Self-Organizing Map (SOM) method based on similarity between species compositions over time. ASFs were determined to be 25 days at Cluster 2 (June-July 2013) and 13 days at Cluster 5 (February-April 2014), whereas no specific ASFs were found at Cluster 1 (April-May 2013), 3 (August-November 2013) (>30 days) and Cluster 4 (December 2013 - January 2014) (<1 day). ASFs showed dramatic seasonality and were negatively related to hydrological wetness conditions, suggesting that sampling interval should be reduced with increasing catchment wetness. A key implication of our findings for freshwater management is that long-term bio-monitoring protocols should be developed with the knowledge of tracking algal temporal dynamics with an appropriate sampling frequency.

Global freshwater resources are widely affected by multiple stressors, such as stream morphological change, point and diffuse sources^1^, which has led to widespread bio-monitoring and bio-assessment. As diatoms strongly respond to environmental changes^2^,^3^,^4^,^5^,^6^, they are increasingly being employed as bio-indicators to assess ecological conditions in lentic and lotic ecosystems around the world^7^,^8^,^9^. Diatoms are a key component of riverine algae and are often the most important primary producers of stream ecosystems^4^,^6^,^10^,^11^,^12^. They are preferred as bio-indicators for a range of reasons besides their sensitivity to subtle changes in environmental conditions due to their short life cycle^2^. For instance, diatoms can be observed in nearly every aquatic environment including fresh- and marine waters, moist terrestrial habitats, such as soils, rock surfaces or epiphytes^13^. They are cosmopolitan, with a wide geographical distribution and well-known autecology of most species^14^,^15^. They are also easy to sample and induce minimal impact on resident biota during collections. Furthermore, their small cell sizes (commonly between 10–200 μm in diameter or length)^16^ allow them to behave similarly to silt particles in stream bodies^17^ and easily be transported by water when a rainfall event occurs, making them a potential tracer for hydrological processes^18^.

Research on the factors controlling the abundance and distribution of the diatom community in streams and rivers has thus become an important part of monitoring overall water quality^19^. Generally the abundance of algal communities is controlled by resource availability (e.g. light and nutrients), disturbances and grazing^6^,^20^. More specifically, studies on the effect of resources and disturbances on diatom communities show that resource factors predominantly govern the processes of biomass gain^21^, while the processes of biomass loss are determined primarily by hydraulic factors^22^. Lange *et al*.^10^ found that light, nutrients and grazing interact to determine stream diatom community composition and functional group structure^10^. For example, compared to ‘low profile’ taxa, ‘high profile’ and ‘motile’ taxa became more prevalent at higher resource levels and the benefit of nutrient enrichment for ‘motile’ diatoms was higher at ambient than at reduced light^10^. Disturbances, for instance caused by extreme fluctuations in stream flows, can also change stream algal communities and functional traits^23^,^24^.

One of the basic premises of diatom-based monitoring is that the species assemblage has been characterized accurately within the study system across spatial and temporal scales. Up to now, temporal dynamics of diatom communities have been widely investigated, since these variations can affect ecosystem processes, functioning and stability, and reflect major shifts in environmental conditions^25^,^26^. Nevertheless, the temporal sampling intervals across these studies varied greatly (sub-daily to yearly) and mainly depended on the research objectives and spatial sampling scales. For large spatial sampling studies, the researchers normally visited the sampling sites only once (few of them revisited for a second time)^23^,^27^,^28^,^29^. Seasonal and/or monthly collections have often been applied at small spatial-scale sampling or in regular bio-monitoring projects^6^,^25^,^30^,^31^. Few studies have applied weekly sampling frequencies^32^,^33^. However, sampling at a daily or sub-daily interval is rare in previous studies^34^. Based on this literature review we raised the following questions:

1. How well does a single sample of diatoms represent the time or season of sampling for comparison of diatom communities among multiple sites?
2. What would be the appropriate sampling frequency (ASF) in order to sample and describe the actual riverine diatom communities over time?

To our knowledge, these two questions are yet unanswered and no studies have determined the ASF of diatom communities. Only with suitable sampling frequency we will obtain enough information about community dynamics and at the same time avoid unnecessary duplication of efforts for lab processing and microscopic identification.

In this study, we used riverine pelagic diatom communities as a model system to answer these two questions and fill this research gap. We collected and analyzed the daily samples of riverine diatom communities over a 1-year period (25 April 2013–30 April 2014) at the outlet of a German lowland river (Fig. 1). The objectives were to 1) describe the temporal dynamics of diatom community, and 2) quantify the appropriate sampling frequency (ASF) at different hydrological conditions.

## Results

### Taxonomic composition and diversity

During the study period (April 2013–April 2014), we observed a total of 113 diatom species belonging to 45 genera (e.g., Achnanthes, Eunotia, Gomphonema, Navicula, Surirella, Ulnaria, etc.; Supplementary material). Within all samples, *Achnanthidium minutissima* and *Navicula lanceolata* were the most abundant species (relative abundances: 39.9% and 15.9% of the total abundance, respectively). Other dominant species with relative abundance >1% were *Planothidium lanceolatum* (6.4%), *Stephanodiscus hantzschii* (4.9%), *Navicula gregaria* (4.8%), *Cocconeis placentula* (3.6%), *Cyclotella meneghiniana* (3.2%), *Nitzschia amphibia* (2.5%), *Diatoma mesodon* (1.7%), *Nitzschia adamata* (1.7%), *Ulnaria biceps* (1.6%), *Punctastriata lancettula* (1.2%), *Frustulia viridula* (1.1%) and *Gomphonema olivaceum* (1.0%). The mean diatom density across the whole sampling period was 1.15*10^6^ cell/L, ranging from a minimum of 1.97*10^4^ to a maximum of 6.36*10^6^ cell/L. The mean values (±SD; min-max) of species richness, evenness, Simpson diversity index, Shannon-Wiener index across all samples were 21 (±6.13; 7–36), 0.65 (±0.17; 0.24–0.96), 0.71 (±0.17; 0.19–0.93) and 1.94 (±0.52; 0.54–2.95), respectively.

### Classification and visualization of sampling dates

Based on the Kohonen Self-Organizing Map (SOM) hierarchical cluster analysis, we classified the samples of 348 days (25 April 2013–30 April 2014) into five SOM clusters (Fig. 2). With 31 exemptions (15 in Cluster 4), SOM Clusters 1–5 were composed mainly of samples taken from April-May 2013, June-July 2013, August-November2013, December 2013 - January2014, and February-April 2014, respectively. To facilitate the following calculation of the appropriate sampling frequency (ASF), we manually categorized exemptions to the closest time groups. For example, although sampling dates (on 15 March 2014, 17 March 2014, 22 March 2014 and 01April2014) were classified into Cluster 3, we manually put these dates into Cluster 5 for the above reason. Nevertheless, MRPP analyses indicated significant differences among and between all different SOM clusters (*p* < 0.01) (Table 1).

Using antecedent precipitation indices (APIs) to indicate catchment wetness conditions, we found that Clusters 1–3 were in low wetness conditions (mean APIs: 17.7, 13.6 and 12.7, respectively), Cluster 4 was in high wetness condition (mean API: 91.0) while Cluster 5 was in moderate condition with a mean API of 43.1 (Fig. 3).

### Appropriate sampling frequency (ASF)

Relationships between percentage similarity index (PSI) and sampling intervals at different SOM clusters showed, as expected, that PSIs decreased with increasing sampling intervals (Fig. 4). We found gradual PSI declines at Cluster 2 (June-July 2013) and Cluster 5 (February-April 2014) with ASFs of 25 days and 13 days, respectively (Fig. 4B,E). We were not able to quantify the ASFs of Cluster 1 and 3, which were >30 days (Fig. 4A,C). In contrast, the ASF of Cluster 4 was <1 day (Fig. 4D). ASF was negatively correlated with API (Fig. 5). During the low and moderate wetness conditions, ASFs vary greatly. For example, an ASF of 25 days would be suitable when API = 13.6 (Cluster 2). However, we found ASFs >30 days at API = 12.7 (Cluster 3) and API = 17.7 (Cluster 1).

## Discussion

The riverine diatom community showed a dramatic temporal dynamic in this study, in accordance with previous studies^4^,^6^,^8^,^35^,^36^,^37^. Although the relationship between riverine algae community variations and environmental factors (e.g., resources, disturbances, grazers, etc.) has been intensively investigated, there is still no general consensus as to which factors regulate riverine algae community in lotic habitats, and furthermore the contributions of main environmental factors to algae variation remain controversial^6^,^38^. The lack of consensus between studies might be due to the fact that the community-environment relationship is dependent on spatial scale. For example, studies at large scale (e.g., national scale) often found that the geographical topography (e.g., altitude, latitude, longitude) and climate were the dominant factors regulating diatom variation^11^,^27^,^29^,^39^. In contrast, at small scales the main regulating factors have been shown to be typically hydrological factors (e.g., discharge), habitat (e.g., substrate composition, sediment input and transport), water quality (e.g., nutrient, dissolved oxygen) as well as bio-interaction (e.g., grazing, competition, parasitism)^27^,^32^.

In this study, we found a close relationship between riverine diatom communities and hydrological conditions (as indicated by antecedent precipitation indices, APIs) as expected (Figs 2 and 3). Given that temperature is an important environmental factor governing algal growth, we might expect that the diatom communities have the potential to change much more quickly during the warmer period than during winter. However, our results showed a contrary phenomenon: PSI community similarities in winter were much lower than those in summer (Fig. 4), indicating the greater contribution of hydrological factors than physicochemical variables to diatom community changes. Hydrological conditions and precipitation are general factors that determine the habitat and affect (directly or indirectly) many other environmental variables that are key factors in diatom community development, such as discharge, residence time, temperature, light availability, and dissolved oxygen^25^,^32^. Moreover, strong precipitation and associated surface run-off may accelerate the transportation of diatom species from soil surface to stream water via soil macro-pores or surface runoff^18^. This process also potentially affects the composition of stream diatom communities. Martínez-Carreras *et al*.^18^ found that the contribution of aerial diatoms in stream water samples collected during rainfall events increased with runoff during all seasons^18^.

Furthermore, storms have been identified as important for the delivery of major nutrients (e.g., P and N) from diffuse agricultural sources^40^, which were a primary factor contributing to variation in phytoplankton assemblages^32^. As a comprehensive index, API integrates not only hydrological processes but also the delivery of nutrients as well as toxic substances such as pesticides and runoff from roads. It can thus be a promising proxy for a large range of environmental factors (e.g., nutrients, hydrological factors) influencing diatom communities. Parallel research (Sun *et al*. unpublished data) found that API was significantly correlated (Pearson, p < 0.05) with chemical variables (e.g., total phosphorus, orthophosphate phosphorus, nitrite-nitrogen, chloride, silicon, sulphate) and hydrological factors (e.g. precipitation, surface runoff, water level) in the study area.

Although diatom communities have been greatly employed in bio-assessment programs, the basic and critical prerequisite is to track their temporal dynamics accurately. This prerequisite is widely recognized but has not yet been studied until now. Recent research carried out in a Chinese subtropical mountain river network has concluded that long-term and high-frequency (i.e., monthly) bio-monitoring protocols should be developed for accurately capturing the benthic algal temporal dynamics^25^. Prompted by the importance of bio-monitoring studies and the scarcity of investigations on ASFs, this study took a step further based on a valuable daily sampling regime and quantified the ASFs at different wetness conditions (indicated by APIs) (Figs 4 and 5). Considering the ASF variation at low and moderate wetness conditions (Fig. 5), we recommend an ASF of 7 days (i.e. weekly sampling) for low and moderate wetness periods when API <63.4, and during high wet conditions (API > 63.4) an ASF of daily or sub-daily (e.g., rain-event based sampling) would be necessary to capture the short-term variation of diatom communities.

Currently, the sampling frequencies recommended by commonly used bio-assessment protocols such as the U.S. Environmatal Protection Agency are mostly single or seasonally sampling depending on the project goals^41^. In addition, some small spatial scale sampling or regular bio-monitoring projects usually apply seasonal or monthly frequencies^6^,^25^,^30^,^31^, while few studies with weekly sampling frequency^32^,^33^. By comparison, our results, provide a concrete baseline for the first time and will be beneficial for future planning of diatom based bio-monitoring campaigns.

Some uncertainties from this study need further investigations. Firstly, our conclusion was drawn from a rough estimation because of the low number of ASFs (Fig. 5). To overcome the above drawback and for safety reasons, the solution in this study was to take a much smaller ASF value than the lowest boundary. Nevertheless, as for the bio-assessment especially for long-term monitoring campaigns, this solution is not ideal, since it might waste a great deal of efforts by potentially taking some unnecessary samples. In future studies, therefore, we should try to increase the number of ASFs, which will allow more accurate ASFs to be developed. In particular, more attention should be paid to the very low wetness condition since the ASFs may increase exponentially (rather than linearly) when API gets close to 0. On the other hand, very low wetness conditions may also pose the risk of stream drying, which is not the case in our study region but requires higher sampling frequency during rewetting. Secondly, the ASFs were computed from the Kielstau catchment with specific climate and precipitation types, and may change if we implement these ASFs into other catchments or climate zones directly, because APIs are catchment-specific, and the actual precipitation related to a certain API will vary between catchments. Thus, further testing and assessment of the applicability of ASFs obtained from this study are still needed. Thirdly, the issue we highlighted may not be limited to only diatom or riverine algae communities. This consideration should also be taken into account for other organisms often used as bio-indicators, such as zooplankton, macroinvertebrates, fish, and macrophytes. Nevertheless, ASFs may be dependent on species generation times and mobility, which should be taken into consideration in future research. Organisms with shorter generation times and higher species mobility might need higher ASFs compared to those with longer generation times and lower mobility. Organism-based stream bio-assessment would be greatly benefited from further studies spreading this approach to other organisms and quantifying the appropriate sampling frequency in the target study reaches with habitat heterogeneities.

In conclusion, the temporal dynamics of the diatom community in our study area were remarkable and the similarity between samples decreased with increasing sampling interval. The temporal dynamic showed a close relationship with hydrological conditions (indicated by APIs) and ASFs were clearly negatively correlated with hydrological wetness conditions. To effectively capture variation processes of diatom assemblages, we recommend a higher sampling frequency (i.e., daily or sub-daily sampling) during high wetness condition when API > 63.4 than during low and moderate conditions when API <63.4 (i.e. weekly sampling). Moreover, we expect that the current approach will ultimately draw the attention of ecologists especially when they prepare long-term bio-monitoring protocols.

## Methods

### Description of the study area

**Figure 1:**
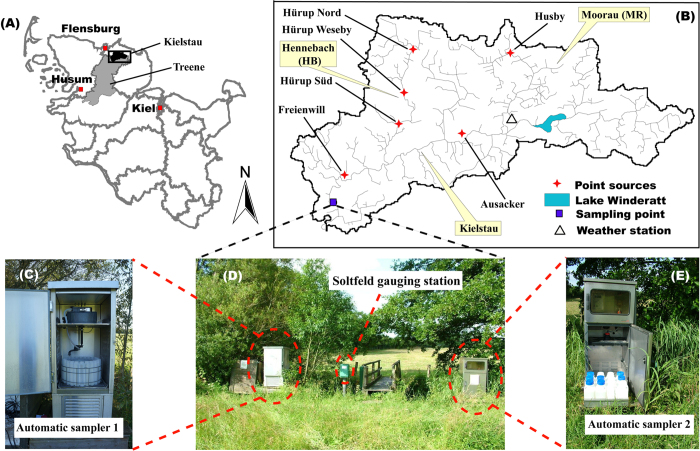
The location of the Kielstau catchment (**B**) in Schleswig-Holstein state (**A**) and three photos of the Soltfeld gauging station and automatic water samplers for daily mixed samples (**C–E**). The maps (**A**,**B**) are created using ArcGIS 10.3.1 software (http://www.esri.com/software/arcgis) and modified with Adobe Photoshop CS6. Photos were taken by Sun (2015).

**Figure 2:**
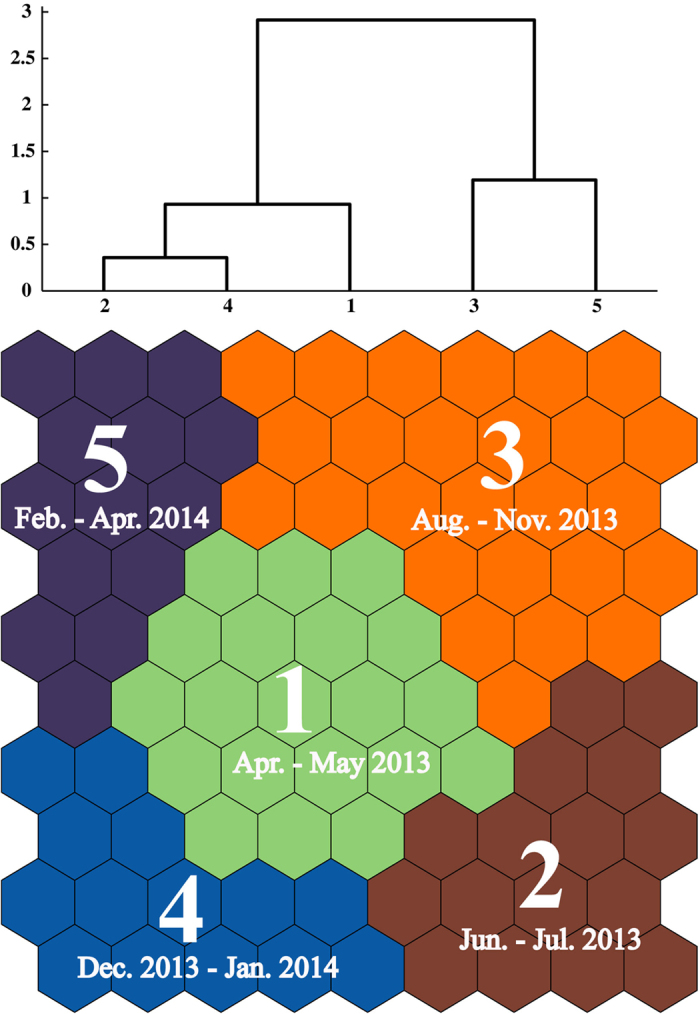
Relationship between each cluster (above) and visualization of the temporal pattern of riverine diatom communities by self-organizing maps (SOMs) (below). Numbers indicate the different clusters. Dates below the numbers are the samples related to each cluster.

**Figure 3:**
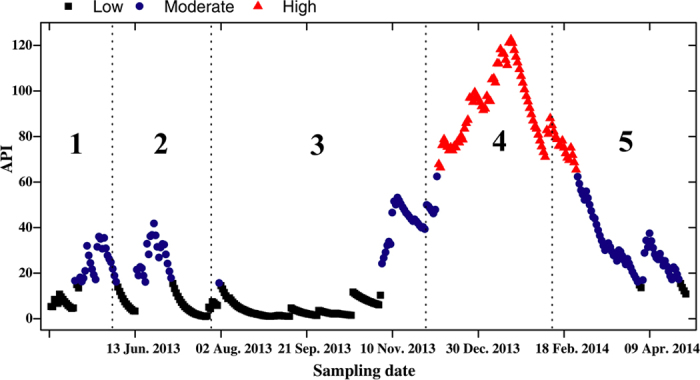
Temporal dynamics of API (antecedent precipitation index) with relation to different SOM clusters (indicated by the large central numbers). To clearly show the wetness conditions we classified the whole APIs by quartiles of a long-term daily APIs (October 2010-June 2015): low: < = 1^st^ quartile (API: 15.5); moderate: 1^st^–3^rd^ quartile (API: 15.5–63.4); high: > = 3^rd^ quartile (API: 63.4). Black squares indicate low wetness conditions, blue circles are moderate, and red triangles are high. Vertical dashed lines separate different SOM clusters.

**Figure 4:**
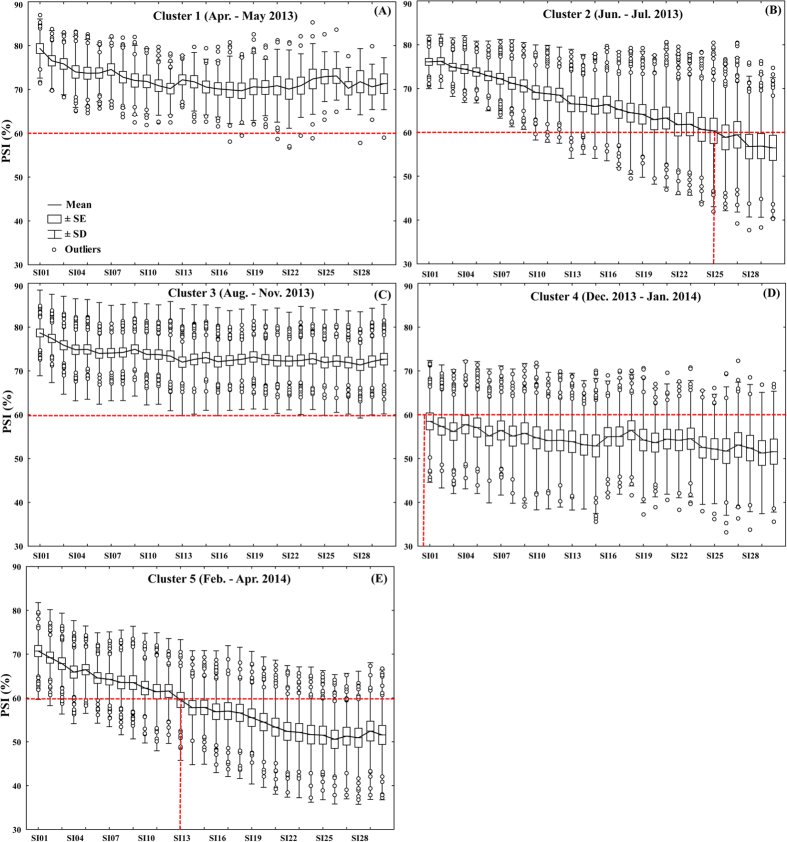
Percentage similarity index (PSI) with different sampling intervals (SI) at different SOM clusters (**A**–**E**). SE = standard error, SD = standard deviation. Red dashed lines are the critical threshold of PSI = 60%.

**Figure 5:**
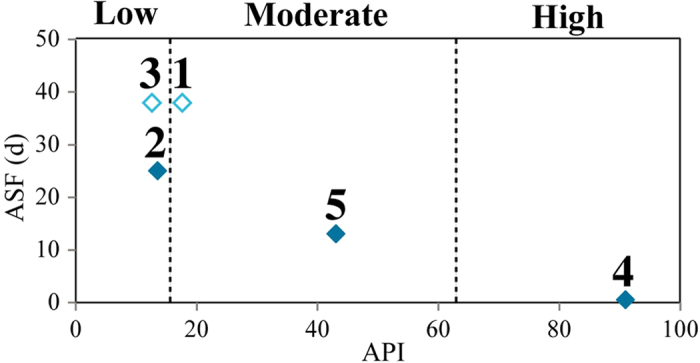
Relationship between ASFs (appropriate sampling frequencies) and APIs (antecedent precipitation indices). Numbers indicate different SOM cluster (Cluster 4 was assigned with 0.5: for details see main text). API indices were mean values of each SOM cluster. Vertical dashed lines separate different wetness conditions (i.e. low, moderate, high). Clusters 1 and 3 (indicated by open diamond) were >30 days.

The Kielstau catchment, a lowland watershed with a drainage area of 50 km^2^, is an UNESCO Demosite for Ecohydrology since 2010 and is located in the northern part of Germany (Schleswig-Holstein). It originates in the upper part of Lake Winderatt and is an important tributary of the Treene River (Fig. 1A). Moorau (MR) and Hennebach (HB) are two main tributaries within the catchment. The drained fraction of agricultural area in the Kielstau catchment is estimated to be 38%^42^. Sandy, loamy and peat soils are characteristic of the catchment. Land use is dominated by arable land and pasture (~55% and ~26%, respectively, of the catchment area)^42^. Six wastewater treatment plants are located in the Kielstau watershed (main stream: Ausacker and Freienwill; Moorau: Husby; Hennebach: Hürup Nord, Hürup Weseby and Hürup Süd) (Fig. 1B). The instream water quality has been considerably influenced by the combination of point sources, artificial drainage systems and diffuse sources in the catchment^4^.

### Sampling methods and primary procedures

The Soltfeld gauging station, installed at the outlet of the Kielstau catchment (Fig. 1D), is a part of the official gauging network of the Federal State of Schleswig-Holstein. Close to the gauging station are the automatic devices for the collection of mixed daily water samples, which were taken during the period from 25 April 2013 to 30 April 2014. With the automatic sampler (Fig. 1C,E), mixed daily water samples were collected and taken back once a week (normally on Monday) to the lab of the Department of Hydrology and Water Resources Management of Kiel University. We expect that algae present in water samples are a mix of suspended benthic algae, and any phytoplankton communities entered the stream from upstream lakes or wetlands or communities developing in the river. Here we will call this the riverine algae community. Moreover, since diatoms are the dominant algal group in many rivers, including the Kielstau where we performed this study^6^, we focus on diatom communities in the following analyses. In the lab, the water samples with known volumes (normally 2.5 L) were fixed in 5% non-acetic Lugol’s iodine solution^43^. Forty-eight hours later, the supernatant liquid was removed and the retained organisms were transferred into glass containers. After sedimentation again, samples were concentrated to 25 mL for further processing. Sampling was not possible on 23 dates because of low temperatures or other technical factors, and a total of 348 samples were collected.

### Diatom preparation and identification

Permanent diatom slides were prepared after oxidizing the organic material by the Hydrogen Peroxide Method (30% H_2_O_2_ solution) and mounted in Naphrax (Northern Biological supplies Ltd., UK, R1 = 1.74) following Biggs and Kilroy^44^. A minimum of 300 valves were counted for each sample using a Zeiss Axioskop microscope at 1000× under oil immersion. Diatoms were identified to the lowest taxonomic level possible (mainly species level) according to following key books^13^,^45^,^46^,^47^. Diatom densities were expressed as cell/L.

### Data analyses

To describe the diatom community, we calculated the diversity indices (i.e. species richness, evenness, Simpson diversity index, Shannon-Wiener index by *diversity* function in R package *vegan*), diatom density and relative abundances of dominant species.

Diatom assemblages were classified using Kohonen Self-Organizing Map (SOM), which is a novel approach to visualize high-dimensional data with unsupervised learning rules^48^. Within the algorithm, the complex statistical relations between high-dimensional data items are converted into simple geometric relationships on a low-dimensional display preserving the most important information from the primary data^49^. In this study, the SOM model described the patterns of temporal variation in diatom species: a total of 113 species were included. Sampling dates with similar species composition and structure were classified into the same neuron or into neighboring neurons, according to the degree of dissimilarity. The output of the SOM was a total of 90 neurons (virtual units), which were arranged into a 10*9 hexagonal lattice to provide better visualization. The map size was set according to a heuristic equation (5n^½^)^50^, and then based on the minimum best values of quantization and topographic errors. The cells of the map were then subdivided into different groups according to the similarity of the weight vectors of the neurons using Ward’s linkage method with Euclidian distance^48^. The group numbers were mainly based on the degree of dissimilarity of each SOM cell in the hierarchical clustering. The unified distance matrix (U-matrix)^51^ and Davies-Bouldin index^52^ were also applied to reinforce the group definition. We carried out all these analyses with Matlab software (Mathworks Inc 2001) using the SOM toolbox^53^. To assess the effectiveness of the hierarchical clustering on the SOM units, we used the Multi-Response Permutation Procedure (MRPP, *mrpp* function in R package *vegan*) to determine whether there was a significant difference between the clusters. We tested null hypothesis that there was no difference among the groups with a Monte Carlo randomization procedure with 999 permutations.

To determine an appropriate sampling frequency (ASF), we calculated the community similarity for each pairing of all possible successive days of 1–30 days within each SOM cluster. We hypothesized that the similarities will decrease with sampling interval increasing (e.g., the similarity between Day_i_ vs. Day_i+1_ is higher than those of Day_i_ vs. Day_i+2_, Day_i_ vs. Day_i+3_, and so on). In this study, the Percentage Similarity Index (PSI) was used as follows:


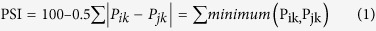


where the PSI represents the similarity between communities i and j, and ranges from 0.0 to 100, with 100 meaning the two communities have identical composition. P_ik_ and P_jk_ are the proportions of individuals present in communities i and j, respectively, that comprise the k^th^ species. In other words, the PSI is the sum of the lowest percent value of a species between communities i and j. In spite of its simplicity, the percentage similarity measure is one of the better quantitative similarity coefficients available^54^,^55^. However, there is still no statistical method which can test the significance of the PSI; thus some scientists suggest a “cut-off” based on mean values from a large number of comparisons to indicate an ecologically-relevant change. For example, Matthews (1998) used the movement or displacement of points over time in ordination space to estimate the amount and directionality of community change. He recommends that a PSI of >0.6 among samples indicates a stable or similar community^56^. Therefore, in this study we also employed this empirical PSI of 60% as the threshold to quantify the ASFs at different SOM clusters.

To estimate the hydrological condition in the catchment, we calculated the API (antecedent precipitation index) on a daily basis (for details, see Fedora and Beschta^57^ and Shaw^58^).


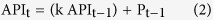


where API_t_ = antecedent precipitation index (mm) at day_t_, and P_t−1_ = precipitation (mm) at the day_t−1_. The precipitation data are from our weather station in Moorau tributary (see Fig. 1). The value of k expresses moisture loss and varies seasonally, usually between 0.85 and 0.98, and is dependent on the potential loss of moisture (mainly through evapotranspiration)^58^. In this study, to estimate k, we used Penman potential evaporation derived from historical data from the University of Keele (Table 11.1 of Shaw^58^).

## Additional Information

**How to cite this article**: Wu, N. *et al*. Importance of sampling frequency when collecting diatoms. *Sci. Rep.* **6**, 36950; doi: 10.1038/srep36950 (2016).

**Publisher’s note**: Springer Nature remains neutral with regard to jurisdictional claims in published maps and institutional affiliations.

## Supplementary Material

Supplementary Information

## Figures and Tables

**Table 1:** Multi-Response Permutation Procedure (MRPP) analyses between different SOM clusters.

	Cluster 1	Cluster 2	Cluster 3	Cluster 4
Cluster 1				
Cluster 2	0.3362 (0.001)			
Cluster 3	0.1595 (0.001)	0.4005 (0.001)		
Cluster 4	0.1090 (0.001)	0.2819 (0.001)	0.2572 (0.001)	
Cluster 5	0.2697 (0.001)	0.2217 (0.001)	0.3259 (0.001)	0.2060 (0.001)

Numbers are A values. P values are listed in the parentheses. The difference among the five clusters is also significant (A = 0.3834, p = 0.001).
