# Supplementary material for: Importance of sampling frequency when collecting diatoms
Source: Sci Rep. 2016 Nov 14;6:36950. doi: 10.1038/srep36950 (PMC5107912; doi:10.1038/srep36950)
Supplement: Supplementary Information [file srep36950-s1.doc]

**Supplementary material:**

# Importance of sampling frequency when collecting diatoms

[[1]](#footnote-2) 

Naicheng Wu ^1,2,3*^, Claas Faber^1^, Xiuming Sun^1^, Yueming Qu^1^, Chao Wang^4^, Snjezana Ivetic^5^, Tenna Riis^3^, Uta Ulrich^1^ and Nicola Fohrer^1^

*^1^Department of Hydrology and Water Resources Management, Institute for Natural Resource Conservation, Kiel University, 24118 Kiel, Germany*

*^2^Aarhus Institute of Advanced Studies, Aarhus University, Høegh-Guldbergs Gade 6B, 8000 Aarhus C, Denmark*

*^3^Department of Bioscience, Aarhus University, Ole Worms Allé 1, 8000 Aarhus C, Denmark*

*^4^Pearl River Fisheries Research Institute, Chinese Academy of Fishery Science, 510380 Guangzhou, China*

*^5^Marine Science and Engineering, Red Sea Research Centre, King Abdullah University of Science and Technology, Thuwal 23955-6900, Saudi Arabia*

**Supplementary table:** List of dominant diatom species with relative abundance of >0.1% in the study area of Kielstau cachment in northern Germany.

| Taxa with relative abundance (%) | | Taxa with relative abundance (%) | |
| --- | --- | --- | --- |
| *Achnanthidium minutissima* | 39.84 | *Navicula menisculus* | 0.16 |
| *Amphora copulata* | 0.15 | *Navicula Rhynchotella* | 0.69 |
| *Amphora ovalis* | 0.26 | *Navicula tripunctata* | 0.24 |
| *Asterionella formosa* | 0.39 | *Nitzschia adamata* | 1.69 |
| *Aulacoseira granulata* | 0.23 | *Nitzschia amphibia* | 2.50 |
| *Cocconeis placentula* | 3.61 | *Nitzschia dissipata* | 0.19 |
| *Cyclotella meneghiniana* | 3.23 | *Nitzschia recta* | 0.37 |
| *Diatoma mesodon* | 1.76 | *Nitzschia sigma* | 0.32 |
| *Encyonema minutum* | 0.11 | *Planothidium frequentissimum* | 0.55 |
| *Fragilaria capucina* | 0.47 | *Planothidium lanceolatum* | 6.37 |
| *Fragilaria crotonensis* | 0.83 | *Pleurosigma delicatulum* | 0.11 |
| *Frustulia viridula* | 1.11 | *Pseudostaurosira parasitica* var. *subconstricta* | 0.17 |
| *Gomphonema olivaceum* | 1.04 | *Punctastriata lancettula* | 1.21 |
| *Gomphonema parvulum* | 0.38 | *Staurosira construens* | 0.47 |
| *Hippodonta capitata* | 0.59 | *Staurosirella martyi* | 0.31 |
| *Melosira varians* | 0.29 | *Stephanodiscus hantzschii* | 4.91 |
| *Meridion circulare* | 0.59 | *Surirella angusta* | 0.35 |
| *Navicula cincta* | 0.20 | *Surirella lacrimula* | 0.33 |
| *Navicula gregaria* | 4.78 | *Ulnaria acus* | 0.20 |
| *Navicula lanceolata* | 15.85 | *Ulnaria biceps* | 1.58 |

1. ^*^Corresponding author: [nwu@hydrology.uni-kiel.de](mailto:nwu@hydrology.uni-kiel.de) [↑](#footnote-ref-2)
